# An analysis of equine round pen training videos posted online: Differences between amateur and professional trainers

**DOI:** 10.1371/journal.pone.0184851

**Published:** 2017-09-18

**Authors:** Erin Kydd, Barbara Padalino, Cathrynne Henshall, Paul McGreevy

**Affiliations:** 1 Faculty of Veterinary Science, University of Sydney, Sydney, NSW, Australia; 2 Department of Veterinary Medicine, University of Bari, Bari, Italy; 3 School of Animal and Veterinary Sciences, Charles Sturt University, Wagga, NSW, Australia; 4 Hillydale Equine, Inverary Road, Bungonia, NSW, Australia; Universidade do Porto Instituto de Biologia Molecular e Celular, PORTUGAL

## Abstract

Natural Horsemanship is popular among many amateur and professional trainers and as such, has been the subject of recent scientific enquiry. One method commonly adopted by Natural Horsemanship (NH) trainers is that of round pen training (RPT). RPT sessions are usually split into a series of bouts; each including two phases: chasing/flight and chasing offset/flight offset. However, NH training styles are heterogeneous. This study investigated online videos of RPT to explore the characteristics of RPT sessions and test for differences in techniques and outcomes between amateurs and professionals (the latter being defined as those with accompanying online materials that promote clinics, merchandise or a service to the public). From more than 300 candidate videos, we selected sample files for individual amateur (n = 24) and professional (n = 21) trainers. Inclusion criteria were: training at liberty in a Round Pen; more than one bout and good quality video. Sessions or portions of sessions were excluded if the trainer attached equipment, such as a lunge line, directly to the horse or the horse was saddled, mounted or ridden. The number of bouts and duration of each chasing and non-chasing phase were recorded, and the duration of each RPT session was calculated. General weighted regression analysis revealed that, when compared with amateurs, professionals showed fewer arm movements per bout (p<0.05). Poisson regression analysis showed that professionals spent more time looking up at their horses, when transitioning between gaits, than amateurs did (p<0.05). The probability of horses following the trainer was not significantly associated with amount of chasing, regardless of category. Given that, according to some practitioners, the following response is a goal of RPT, this result may prompt caution in those inclined to give chase. The horses handled by professionals showed fewer conflict behaviours (e.g. kicking, biting, stomping, head-tossing, defecating, bucking and attempting to escape), and fewer oral and head movements (e.g. head-lowering, licking and chewing) than those horses handled by amateurs Overall, these findings highlight the need for selectivity when using the internet as an educational source and the importance of trainer skill and excellent timing when using negative reinforcement in horse training.

## Introduction

Natural horse (NH) training methods are popular among amateur and professional horse trainers [1] and have been the subject of recent scientific enquiry [2,3,4]. One school of thought is that NH methods are more humane and result in a better human-horse relationship than other methods [2,5]. However, there is considerable variation in the specific training techniques applied by NH trainers [1,6,7,8,9,10] and in the descriptions they attract in the scientific literature [2,5,11,12,13].Nonetheless, common to all the NH methods reported in the scientific literature is the use of a training technique known as the “chase-away” [4,7,14] which takes place in a circular pen known as a round pen.

The use of a round pen to facilitate training of horses dates back to ancient Roman times, when the so-called gyrus was used to train horses [15]. In contemporary round pen training (RPT), the horse is initially chased away from the handler by the application of aversive stimuli such as arm-waving, rope throwing and vocal cues. After a period of flight behaviour such as trotting, cantering or galloping, the intensity of the aversive stimuli is generally reduced. The ultimate goal of the method is to condition the horse to remain close to and follow the trainer (for review, see Henshall & McGreevy, [16]). The removal of the aversive stimulus as soon as the desired response emerges is an example of negative reinforcement [17]. As with any form of negative reinforcement, efficacy relies on the immediate removal of the aversive stimulus [18]. Failure to do so increases the risk of punishing the desired response instead and habituating the horse to the aversive stimulus with the likelihood that the intensity of the stimulus will be need to be increased to elicit the response in the future [19]. Furthermore, welfare implications arise from exposing horses to aversive stimuli from which they can’t escape or avoid [20].

Although RPT is promoted as humane and effective, there are currently limited studies detailing a typical RPT session and the response of horses to this training method [3,4,5,7,8,9,12,21,22,23]. RPT is advocated by professional NH trainers as part of foundation training (i.e., prior to the first saddling and riding of unstarted horses) or to retrain horses with undesirable behaviours [7,8,9,21]. When the training outcomes of experienced RPT trainers were compared with those of conventional or traditional trainers, RPT methods were associated with lower heart rates during the first saddling and first riding [5,12]. However, in the first of these studies [5] only two trainers were compared, meaning that the experience of the trainer and the techniques deployed were potentially conflated. As such, further studies involving more trainers should be conducted to confirm these preliminary findings before the results of this study can be generalised to the wider community. It is worth noting that, to the authors’ knowledge, previous studies have not compared the work of amateur and professional RP trainers, as is the case for the current study.

RPT is frequently advocated on lay forums as a means of resolving a wide range of undesirable equine behaviours from poor leading responses, failing to stand when mounted, bucking and not being caught. Some of these behaviours reflect poor deceleration responses, while others reflect a corrupted acceleration response and others are thought to arise as a result of behavioural conflict [24]. It is difficult to see how one training construct (with the goal of conditioning the horse to follow the human) can address all of these responses. The standard interpretation of RPT outcomes is that the horses comes to recognise the human as an analogue of a dominant horse or leader and thus becomes compliant or submissive in all subsequent human-horse interactions [7]. This interpretation relies on a simplistic analysis of equine social interactions in free-ranging herds in which dominance hierarchies are assumed to be linear and unvarying within all horse herds, both domestic and free-ranging. However, this interpretation is not supported by the data [16]. A recent study has found no evidence of leadership in horse herds as defined by many NH trainers [25] and there is considerable variation in the social organization of horse herds [26]. Consequently, the behaviour observed in one group of horses may not necessarily be representative of all horses and training methods apparently based on such observations may also lack salience for some horses or contexts.

NH trainers sometimes claim that the cues and training outcomes that result from RPT are chiefly of ethological salience and controversy exists over whether the licking and chewing responses that RPT values are signs of submission and acknowledgment of the human as a leader [16]. Behaviours such as licking, chewing and head-lowering and following the trainer are interpreted as intraspecific signalling signifying the horse’s respect for the superior dominance status of the trainer [7]. Furthermore, the reliance of RPT on aversive stimuli to elicit a flight response at the start of training is of concern to some observers [27]. If, in response to the aversive stimuli, the horse fails to perform so-called submissive behaviours, such as following the trainer, or if the trainer fails to observe these responses, the horse may be subjected to aversive stimuli that inadvertently prevent reinforcement of the ultimately desired behaviour. Furthermore, the reliance on putative homologues of the equid ethogram to interpret responses in the round pen obscures the conditioning mechanisms at work [28] and may dissuade less experienced trainers from correctly reinforcing the desired responses. Some owners who have attempted RPT report a range of unwanted behaviours. These reports suggest that, despite the success attested by some, there is considerable variation in the application of RPT techniques and that chasing does not produce a predictable response in all horses. Clearly, trainers’ acquired or natural aptitude for horsemanship can alter training results [29]. Despite the use of RPT in all of the NH studies to date, there are no data on the character and distribution of chasing and the frequency of unwanted equine responses that generally accompany the technique.

Many professional and amateur RPT sessions are posted on the video-sharing website YouTube. RPT sessions are usually split into a series of bouts; each including two phases: chasing/flight and chasing offset/flight offset. Among these sessions, there is considerable variation in duration, intensities, the cues used by trainers and the behaviours elicited from the horses. The aim of this study was to collect baseline data on the characteristics of RPT sessions as posted online, test the difference in the application of RPT between apparent amateurs and professionals (as a gauge of the role of experience) and enhance horse welfare by highlighting the use of the Internet in equitation science. Trainers were divided into amateur and professional according to whether the videos were accompanied by online materials that promoted clinics, merchandise or a service to the public. The study assessed the extent to which RPT, as published online, is humane and a credible form of advice on the technique. The study examined publicly available RPT sessions to establish whether there are common elements of RPT and to identify the sources of variability among RPT sessions. It measured duration of sessions, duration of chasing and not chasing as well as the individual behaviours of the horse and trainer.

## Methodology

The search function of the public video sharing website YouTube (www.youtube.com) was used to source the video clips. All data were collected from this site and the researchers had no contact with any horses or trainers and did not require ethics approval. A YouTube search was conducted for videos using the keywords “round pen training”, “horse training”, “horse taming” and “natural horsemanship” and “round penning” and “join up”. Since people generally post their own videos on YouTube, the videos are expected to be of optimal quality to represent each practitioners’ best practice. Introductory information provided by those posting the videos about whether the videos were accompanied by online materials that promoted clinics, merchandise or a service to the public was used to categorise the trainers into two groups: amateur and professional. If practitioners offered training services to the public, they were considered professional.

The inclusion criteria for videos were that the video was of good quality, the horse was trained at liberty in a round pen, the training session contained more than one bout (one bout being equal to two phases: chasing and chasing offset) and commenced without the trainer first riding or putting a saddle on the horse and, finally, all of the round pen area was visible. The exclusion criteria for videos were that the trainer saddled, mounted or rode the horse, the trainer applied a rope or long rein to the horse (such that it was no longer at liberty) and the training session lasted longer than 400 seconds. The selection of the videos was made in agreement with the selection criteria by three of the research team members. Adherence to the selection criteria was rigorous in order to minimise variation between videos. It should be noted that some variation in the videos was immediately obvious. This was due to individual differences in videos from each poster and included, but was not limited to, differences in area of round pen, materials used to construct the round pen, surface used in RPT, history and age of the horse, the reason the horse was being trained and personal editing choices of the video that may include cut scenes. Each video featured a unique horse/trainer pair, on three occasions (one amateur and two professionals) the same trainer featured in two videos. From more than 300 candidate videos in which RPT was applied, a total of 24 videos of amateurs and 21 videos of professionals satisfied the inclusion criteria and were taken forward for analysis.

After the videos had been categorised, two equine ethologists from the research team, working together, watched all the videos and completed a focal sampling ethogram (see Table 1). To avoid potential biases, as far as possible the trainer’s category was not revealed to the observer until after the data had been collected.

**Table 1:** Behavioural states and events selected in the focal sampling ethogram.

Phase	Event frequency (n)	Definition
**Chasing**	Bite threat	Trying to bite the trainer
	Bucking/shying	All four legs off the ground including kicking out with both hind legs (Waring, 2003)
	Stomping	Rapid lift of the foreleg
	Kicking with one leg	Lifting a single hind leg and rapidly extending it away from the body (Waring, 2003)
	Kicking with two legs	Lifting both hind legs and rapidly extending them away from the body (Waring, 2003)
	Head-tossing	Rapid lateral or vertical movement of the head away from the body (Waring, 2003)
	Change direction	Change in the direction: right toward left, or the reverse, in walk, trot or gallop
	Neigh/snort	Any type of neigh or snort, identified visually (i.e. not using sound)
	Sniffing	The horse sniffs the ground
	Lick and chew/oral movements	Opening of mouth with extension and retraction of tongue, lip smacking without tongue extension, lateral jaw movements involving partial opening of lips (McGreevy, 2004)
	Defecation	Evacuation of large bowel (McGreevy, 2004)
	Escape attempt	Any rapid movement directed toward the fence where the horse looks for a possible escape. This may include the horses chest touching the fence
	Head-lowering	The horse lowers head below the withers
**Non chasing**	Stopping face-toward or side on	Coming to a standstill with the trainer in-line with the forefeet of the horse
	Stopping during following	Coming to a standstill while following the trainer
	Stepping toward (n° of steps)	Individual steps of the forelegs toward the trainer
	Stepping back (n° of steps)	Individual steps of the forelegs backward in the opposite direction of the trainer
	Touching the trainer	Direct contact with the trainer by the nose of the horse, initiated by the horse
	Neigh/snort	Any type of neigh or snort, identified visually (i.e. not using sound)
	Defecation	Evacuation of large bowel (McGreevy, 2004)
	Sniffing	The horse sniffs the ground
	Lick and chew/oral movements	Opening of mouth with extension and retraction of tongue, lip smacking without tongue extension, lateral jaw movements involving partial opening of lips (McGreevy, 2004)
	Head-lowering	The horse lowers head below the withers
	**States duration (sec)**	
**Chasing**	Walking	The horse walks
	Trot	The horse trots
	Canter/gallop	The horse gallops or canters
	Standing/stopping toward fence	The horse stops and stands toward the round pen fence
**Non chasing**	Standing/stopped	The horse stops and stands quietly toward their trainer
	Following	The horse follows their trainer

The number and duration of the bouts were recorded, as was the duration of each chasing and non-chasing phase. The chasing phase of each bout was measured from the period when the trainer used arm movements, rope throwing or other actions to initiate movement in the horse. The end of a chase and the beginning of a non-chase phase was measured from the period when the trainer ceased applying such stimuli resulting in the slowing of the horse. Additionally, the observer recorded if the trainer stopped chasing when the horse was standing/stopped. It is important to note that we are using the term chasing in a purely descriptive sense. It is not meant pejoratively or to imply that the horse can anticipate being caught. The frequency and the duration of selected behavioural events and states (see Table 1) were also recorded for each phase, along with the number of gait transitions the horse made during each chasing bout. The actions of trainers are described in Table 2.

**Table 2:** Ethogram of selected trainer behaviours and states.

Phase	Event frequency (n)	Definition
**Chasing**	Use of aversive stimuli	The trainer chases the horse with whips, ropes or stones
	Kicking sand toward the horse	The trainer kicks some sand toward the horse
	Change position toward the horse	The trainer changes position from angled away from the horse or back towards the horse to front toward the horse
	Arm movements	The trainer raises one or both arms away from body toward the horse without training aids (whip or rope etc.)
**Non chasing**	Looking down	The trainer orientates the head away from the horse and toward the ground or on the angle
	Change position away from the horse	The trainer changes position from front toward the horse to angled away or back towards the horse
	**States duration (sec)**	
**Chasing**	Looking upright	The trainer looks face up to the horse
**Non chasing**	Contact with the horse	The trainer touches the horse

### Statistical analysis

A general weighted regression was used to analyze trainer arm movements per bout, with bout number used as weighting. Poisson regression analysis was used to analyze the time trainer spent looking at the horse and number of transitions, conflict behaviours and time spent in canter or gallop, and number of oral and head movements. The Poisson model was chosen due to the differences in individual video length and in time spent in each gait by any given horse. To assist in analysis, some horse behaviours were grouped together under the term ‘conflict behaviours’. These were biting, bucking, shying, stomping, neighing, snorting, kicking (with one leg or two), head-tossing, defecation and escape attempts. Likewise, behavioural signs of so-called submission were grouped together for analysis under the term oral and head movements (OHM). These were licking, chewing and oral movements, and head-lowering.

## Results

The behaviour of horses and trainers during RPT was recorded for both chasing and non-chasing phases. The distribution and proportion of time spent by horses and trainers of both categories during a RPT session appear in Table 3.

**Table 3:** Descriptive statistics (mean and standard deviation for both professional and amateur trainers) of typical RPT sessions from a study of online videos (n = 45).

	Total	Total SD	Amateurs	Amateur SD	Professionals	Professional SD
*Mean total duration of non-chasing phase*	**107.44**	66.32	**94.54**	57.08	**122.19**	74.16
*Mean total duration of chasing phase*	**143.27**	60.52	**150.96**	65.29	**134.48**	54.81
*Total duration of session*	**250.71**	81.00	**245.50**	82.22	**256.67**	81.18
*Number of bouts*	**3.02**	1.10	**2.79**	1.02	**3.29**	1.15
*Mean number of conflict behaviours exhibited during RPT session*	**2.38**	3.35	**3.04**	4.14	**1.62**	1.96
*Mean number of oral and head movements exhibited during RPT session*	**2.13**	3.75	**3.25**	4.73	**0.86**	1.39

A bout consists of a chasing phase offset by a non-chasing phase. ‘Conflict behaviours’ include biting, bucking, shying, stomping, neighing, snorting, kicking (with one leg or two), head-tossing, defecation and escape attempts. Oral and head movements include licking, chewing and oral movements and head-lowering.

**Fig 1:**
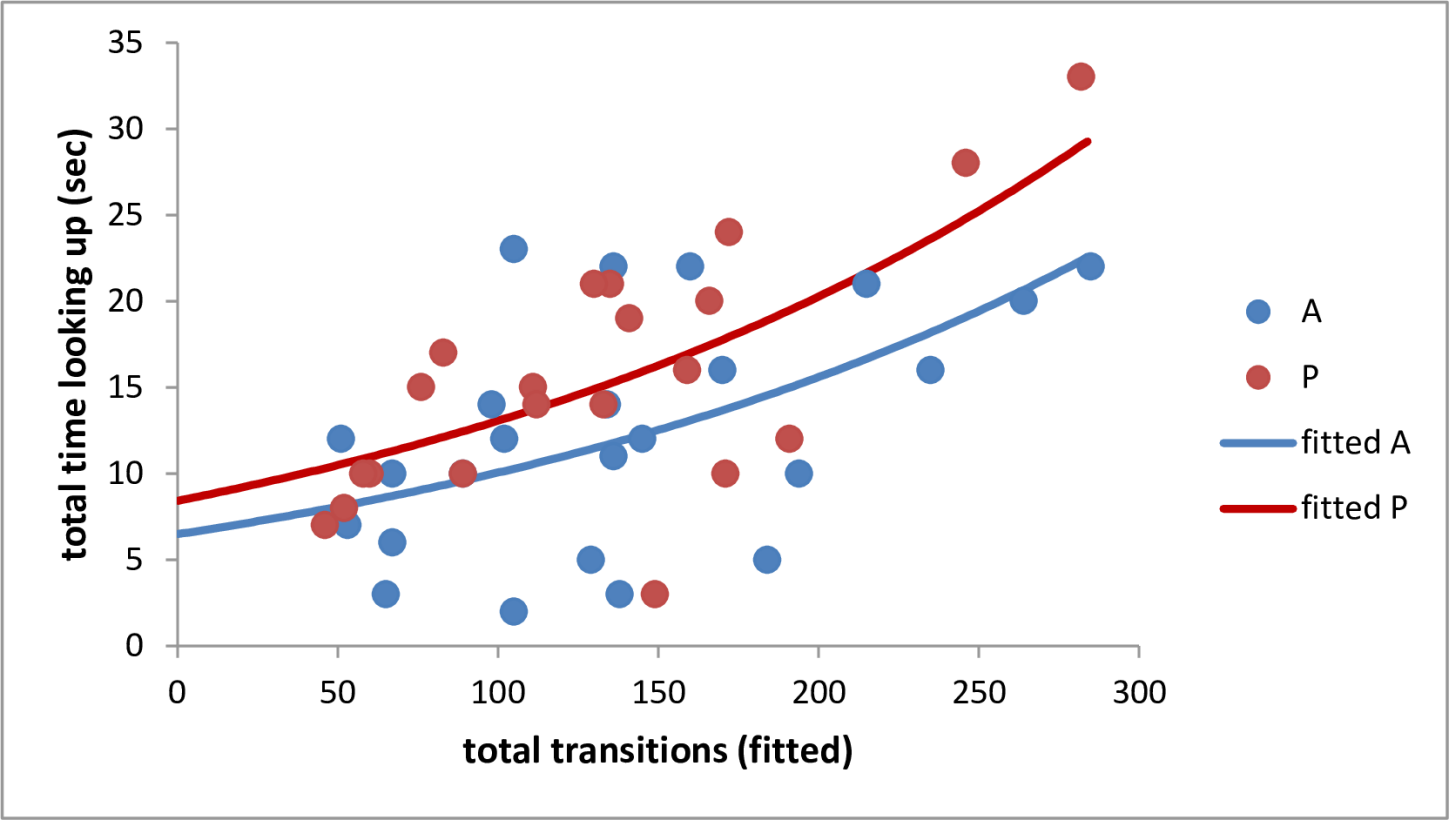
Relationship between total transitions and total time looking up by amateur (A, n = 24) and professional trainers (P, n = 21), with fitted Poisson regression curves.

A general weighted regression analysis, weighted by number of bouts, revealed that, when compared with amateurs, professionals showed fewer arm movements per bout. However, this failed to reach statistical significance (p = 0.204). Poisson regression analysis showed that professionals spent more time looking up at their horses, when transitioning between gaits than amateurs did (Fig 1, p<0.05). Regardless of trainer category, the total time spent looking up at the horse correlated strongly with the total transitions observed (p<0.001). More transitions between gaits were seen as total time spent looking up at the horse increased (Fig 1). The horses handled by professionals showed fewer conflict behaviours at the canter and gallop (p<0.05). Additionally, horses handled by professionals exhibited fewer OHM than those handled by amateurs (p<0.05). Poisson regression analysis showed the probability of horses following the trainer was not significantly associated with amount of chasing, regardless of the category of trainer (p = 0.557).

## Discussion

The results of the study reveal considerable variation in the RPT technique exhibited by trainers who choose to post their sessions on YouTube. Variation among training technique was demonstrated in the chasing phase, along with the trainer’s cues and the resulting behaviour of the horses. These findings have implications for equine welfare and raise questions about the interpretations of equine ethology that NH trainers often rely upon. They also provide a cautionary tale to those intending to use public sources of information from the internet, such as YouTube, as a guide in training their horses. If performed well, RPT can result in effective training. However, unquestioning observers may apply techniques without appreciating the problems they are creating for the horse and the violation of a core principle of ethical equitation: to dissociate fear and avoidance responses [30].

It is important to acknowledge the limitations of this study. Clearly, video records can be edited before being posted, so we must accept that, although we expect these video files to represent the trainers’ best practice, they may present an obscured record of authentic practice. Nevertheless, we should consider them as they are perceived by viewers online. This is important since the educational value of such material is part of our enquiry. Due to the variable quality of audio tracks, the videos were scored with sound off. We accept that this may have overlooked some qualifying remarks by trainers that may, for example, have explained they are showing flaws in their practice to make a certain point.

Notwithstanding the study’s acknowledged limitations, we propose that the difference between amateurs and professionals reported here is of importance. We note that to become professional (in any method), a horse trainer needs first to be an amateur. It would be interesting to undertake the same exercise for alternative training techniques (e.g. conventional training). The current study shows that professionals look up at their horse more than amateurs. This strongly suggests that professionals have better timing and respond to their horse’s behaviour with greater efficacy than amateurs. In light of this, it is probable that trainers who are paying greater attention to their horse are less likely to observe (or create) conflict behaviours. In addition, because NH trainers are looking for the presentation of the oral and head movements, as soon as they are observed they are recognised and training enters the next phase (non-chasing). If amateur trainers do not look at their horses as much as professional trainers (as shown in this study), they have more opportunity to, firstly, get their timing wrong (which may inadvertently trigger increased conflict behaviours), and secondly, to miss when the horse is displaying the critical oral and head movements (whose rate may escalate if they are not acted on by the trainer). Observational skills are critical to the application of cues at moments most conducive to animals’ ability to respond correctly and it is likely that professional trainers observe their horses more closely than amateurs in order to apply cues more precisely. Professional trainers are presumably more experienced than amateurs and, in the light of that experience, are expected to have better timing [17]. It is also possible that the trend reported here towards fewer arm movements per bout seen in professionals, as compared with amateurs, is another indicator of the skill of professional trainers. It is a principle of negative reinforcement that aversive stimuli are minimised to avoid the risk of punishing the desired response or habituating the horse lest an increase in aversiveness is needed in future [19]. In most of the round pen sessions in the current study, chasing phases began with the trainer waving an arm (or a rope) and gazing directly at the horse. Arm-waving should cease after the horse reaches the speed desired by the trainer. As observers, we cannot be sure of what speed or gait the trainer was aiming for, so we have not commented on the precision of cessation of these stimulations. Arm-waving or chasing of the horse that continues after the horse is running has the potential to create a situation where confusion and conflict behaviours result [31].

After a chasing phase, the onset of a non-chasing phase is usually signalled by a postural change by the trainer with an accompanying cessation of eye contact by some but not all trainers (see Anderson and Hendrikson [32] and Roberts [7]). Depending on the horse’s response to training, various shifts in the direction of the gaze are said to communicate either aggressive or affiliative intentions from the trainer with a direct gaze used to drive the horse away [7] or used to cue the horse to approach the trainer [33]. Various empirical studies have explored the effect of gaze direction and human eye contact on horse responses in an array of different contexts with differing outcomes. Seaman et al. [34] exposed 33 horses of mixed age and breed to a single human and reported that eye contact or its absence had no effect on horses’ latency to approach that person. Similarly, Verrill and McDonnell [35] showed that neither an averted nor a direct gaze affected an approaching handler’s ability to catch either semi-feral or well-handled horses (n = 104) in a paddock. By contrast, Birke et al. [36] reported that semi-feral ponies (n = 12) were more likely to flee when the gaze of the handler was directed away from them, thus countering the view that a direct gaze is threatening. Concentrated food is known to trigger heightened arousal in horses [31,37] and so has been used, with some success, to unpick the role of eye gaze in human-horse interactions. For example, using a food test, [38] found that horses (n = 60) were more likely to approach a human who was gazing directly at them than one who was oriented away. Similarly, Proops and McComb [39] showed that horses (n = 36) could be trained to recognise and approach handlers displaying a direct gaze in a food reward test. These reports indicate that horses may observe a handler’s gaze and head orientation but the salience of any discrimination that emerges from these observations reflects operant conditioning more than an innate ethological significance. In other words, eye contact or its absence can, through classical associations, come to signify chase or offset of chasing depending on the practitioners’ choice. The current data simply indicate that professionals chose to spend longer engaging eye contact.

According to the principles of learning theory [17,24,40,41], the chasing phase of RPT should be offset by a period of non-chasing, as the horse begins to follow the trainer. However, it is noteworthy that the current data showed no association between the amount of chasing and the following behaviour. The following of the trainer is presented as one of the primary goals of RPT and posited to indicate that the horse views the trainer as a leader and wishes to remain in the human’s company; valuing it as it might value the safety of a herd of conspecific analogues. It is likely that triggering the horse to follow is simply a trained response developed through negative reinforcement: the correct application of pressure during the chasing phase and the release of pressure on the moment the horse offers the desired behaviour. Indeed, a study conducted by Maros and others [42] indicated that the behaviour of horses following humans was essentially a consequence of training. A recent paper by Muller et al. [14] also questions how well the putative reasons for which a horse in a round pen may follow or approach a trainer align with an ethological framework. Observational studies of RPT often disagree with some of the NH theories previously offered to explain equine motivation. There is no evidence that the following response confirms that the horse views the trainer as a leader or herd member of higher social status. Indeed, the whole question of social order is controversial and its scientific scrutiny is fraught with problems, not least because of the number of variables at play. In the peer-reviewed literature, a number of factors have been identified as determinants of social order in horse herds (see Henshall and McGreevy [16]) and explain why leadership is not necessarily stable [43].

Licking and oral movements in RPT are purported to be a sign of submission from the horse [7] or the horse “thinking” about its training [6]. Unfortunately, these responses are absent from the key published equine ethograms [44,45]. Sighieri et al. [46] described oral movements in subjects (n = 5) exposed to RPT and likewise Krueger [47] reported that licking and chewing were performed by horses during RPT but chiefly while they were approaching the trainer rather than during the chase-away [47]. Oral movements in young horses in round pens with older mares have been noted as occurring most frequently when the young horse is facing away from the mare and are thus unlikely to be a form of signalling between the horses [3]. Although oral movements are understood in NH circles to be ‘submissive’ behaviours, another theory is that oral movements are instead a response to a stressful situation [48] that triggers an adrenaline response that, among various physiological outcomes, dries the buccal mucosa and prompts jaw and tongue movements. A recent study by Nicholls [49] on horse’s behavioural and cardiac responses to having their manes pulled supports this hypothesis with horses performing licking and chewing during mane pulling (thought to be painful), compared to controls. The role of stress in the RPT may explain the observed increase in oral behaviours in horses handled by amateurs. As amateur handlers are likely to be still learning to apply and remove pressure at the optimal time to reinforce the appropriate response, they may be more likely to cause confusion and a consequent increase in stress when doing so and thus prompt more oral behaviours in their horses. Whilst the results of the current study suggest that the occurrence of oral movements was positively correlated with the occurrence of conflict behaviours, further studies are needed to investigate the meaning of oral behaviours in horses during a RPT session.

Conflict responses in horses are those behaviours observed as a result of confusion or fear [24]. They can include various signs from subtle head-shaking and foot-stomping to more dramatic behaviours such as shying, bucking, bolting and biting [24]. It is perhaps not surprising that fewer conflict behaviours were observed in horses with professional handlers compared with those with amateur handlers when horses were cantering and galloping. Again, this seems to confirm that the skill of the handler can have a substantial impact on the emergence of behavioural stress responses of the horse during training.

## Conclusions

This study highlights the need for selectivity when using the Internet as an educational resource. The results reflect important differences in skill and outcomes between amateur and professional trainers as available on the website YouTube, with likely implications for horse welfare.

## Supporting information

S1 Data Supporting information data set. (XLSX)
